# Coping Strategies and Quality of life: Reaction to the COVID-19 Pandemic Among Romanian physicians

**DOI:** 10.1192/j.eurpsy.2023.1246

**Published:** 2023-07-19

**Authors:** R.-M.-A. Stretea, Z. Milhem, A.-I. Forray, C.-A. Crișan

**Affiliations:** 1 Clinical Psychiatry Ward I, Clinical Hospital for Infectious Diseases; 2 Clinical Psychiatry Ward I, ”Octavian Fodor” Institute of Gastroenterology and Hepatology Cluj-Napoca; 3 Public Health and Healthcare Management; 4 Neurosciences, “Iuliu Hatieganu” University of Medicine and Pharmacy, Cluj-Napoca, Romania

## Abstract

**Introduction:**

The COVID‑19 pandemic has raised multiple psychological challenges among most healthcare workers, from anxiety to depression, burnout, sleep disorders, and substance use disorders. Thus, the burden caused by this prolonged medical crisis has inevitably drastically lowered the quality of life of the medical staff. In order to mitigate the negative effects of the pandemic, healthcare workers resorted to various coping strategies, with better or worse outcomes.

**Objectives:**

The present study aims to identify Romanian physicians’ main coping mechanisms and evaluate the role of positive and negative stress-reducing strategies on quality of life.

**Methods:**

A cross‑sectional national survey was conducted using a web-based questionnaire among physicians practicing in Romania (n=265). In addition to socio-demographic and professional information, the questionnaire addressed participants’ coping mechanisms using the COPE inventory and quality of life with the WHOQOL-Brief scale. Descriptive statistics, Pearson correlations, and multiple linear regressions were used in the statistical analysis.

**Results:**

In total, 265 physicians consented to their participation in the survey. Of those who responded, 84.5% identified as female, 92.1% had a permanent residence in a urban setting, 63.8% were married and 55.1% attained a master’s degree, a PhD diploma or equivalent level of education. The results showed that optimism was higher in male professionals, while avoidance coping was higher in female health professionals. The mean values of QoL subscales were: 74.7± 18.3 for the general quality of life, 70.8± 20.7 for health satisfaction, 64.0± 14.2 for the physical area, 61.7± 16.2 for the psychological area, 61.2± 20.3 for the social relationships area and 64.7± 12.7 for the environment area. Specific coping mechanisms (emotional venting, behavioral and mental disengagement) were associated with lower quality of life. In contrast, emotion-focused (positive reinterpretation and acceptance), problem-focused strategies (planning, active coping, suppression of competing activities) and humor were associated positively with most QoL subscales scores.

**Conclusions:**

Our data points to specific protective characteristics and some detrimental factors on physicians’ quality of life during the pandemic, with the implication that these factors may be important considerations for mitigating distress and psychiatric disorders for healthcare workers during times of high stress. Concerted initiatives to improve wellness in healthcare workers ought to develop targeted programs to ensure adequate psychological support.

**Disclosure of Interest:**

None Declared

